# A Novel Socioeconomic Measure Using Individual Housing Data in Cardiovascular Outcome Research

**DOI:** 10.3390/ijerph111111597

**Published:** 2014-11-12

**Authors:** Duk Won Bang, Sheila M. Manemann, Yariv Gerber, Veronique L. Roger, Christine M. Lohse, Jennifer Rand-Weaver, Elizabeth Krusemark, Barbara P. Yawn, Young J. Juhn

**Affiliations:** 1 Department of Pediatric and Adolescent Medicine, Mayo Clinic, 200 First Street SW, Rochester, MN 55905, USA; E-Mails: dukwon.bang@gmail.com (D.W.B.); RandWeaver.jennifer@mayo.edu (J.R.-W.); krusemark.elizabeth@mayo.edu (E.K.); 2 Department of Internal Medicine, Division of Cardiology, Soonchunhyang University Hospital, 22, Daesagwan-gil (657 Hannam-dong), Yongsan-gu, Seoul 140-743, Korea; 3 Department of Health Sciences Research, Mayo Clinic, 200 First Street SW, Rochester, MN 55905, USA; E-Mails: manemann.sheila@mayo.edu (S.M.M.); Gerber.Yariv@mayo.edu (Y.G.); roger.veronique@mayo.edu (V.L.R.); lohse.christine@mayo.edu (C.M.L.); 4 Department of Epidemiology and Preventive Medicine, School of Public Health, Sackler Faculty of Medicine, Tel Aviv University, P.O. Box 39040, Tel Aviv 699780, Israel; 5 Division of Cardiovascular Disease, Department of Internal Medicine, Mayo Clinic, 200 First Street SW, Rochester, MN 55905, USA; 6 Department of Research, Olmsted Medical Center, 210 Ninth Street SE, Rochester, MN 55904, USA; E-Mail: BYawn@olmmed.org; 7 Division of Allergic Diseases, Department of Internal Medicine, Mayo Clinic, 200 First Street SW, Rochester, MN 55905, USA

**Keywords:** socioeconomic status, myocardial infarction, all-cause mortality, health disparities, housing

## Abstract

*Background*: To assess whether the individual housing-based socioeconomic status (SES) measure termed HOUSES was associated with post-myocardial infarction (MI) mortality. *Methods*: The study was designed as a population-based cohort study, which compared post-MI mortality among Olmsted County, Minnesota, USA, residents with different SES as measured by HOUSES using Cox proportional hazards models. Subjects’ addresses at index date of MI were geocoded to real property data to formulate HOUSES (a z-score for housing value, square footage, and numbers of bedrooms and bathrooms). Educational levels were used as a comparison for the HOUSES index. *Results*: 637 of the 696 eligible patients with MI (92%) were successfully geocoded to real property data. Post-MI survival rates were 60% (50–72), 78% (71–85), 72% (60–87), and 87% (81–93) at 2 years for patients in the first (the lowest SES), second, third, and fourth quartiles of HOUSES, respectively (*p* < 0.001). HOUSES was associated with post-MI all-cause mortality, controlling for all variables except age and comorbidity (*p* = 0.036) but was not significant after adjusting for age and comorbidity (*p* = 0.24). *Conclusions*: Although HOUSES is associated with post-MI mortality, the differential mortality rates by HOUSES were primarily accounted for by age and comorbid conditions. HOUSES may be useful for health disparities research concerning cardiovascular outcomes, especially in overcoming the paucity of conventional SES measures in commonly used datasets.

## 1. Introduction

Cardiovascular disease (CVD) is the most common cause of death among adults in the United States, accounting for 500,000–700,000 deaths per year [1]. Among CVD, myocardial infarction (MI) affects nearly 1.5 million people annually in the United States, with an annual incidence rate of 600 per 100,000 persons [2,3]. MI is responsible for major health care expenditure and is a socioeconomic issue (estimated indirect cost in 2013, $202.5 billion) [4].

Impacts of socioeconomic status (SES) on CVD have been widely reported in the United States and other countries [5,6,7,8]. For example, individual SES measures including education, income, occupation, and employment status, have shown to be significantly associated with mortality after MI [9,10]. In promoting clinical research that addresses health disparities in CVD, large-scale administrative datasets derived from medical records have been increasingly utilized [11,12]. These trends will only continue to rise given the foreseeing advancement of medical informatics in the future. However, when clinical researchers utilize administrative datasets for CVD research concerning health disparities, they often encounter obstacles, such as the unavailability of SES measures [13]. Limited data could be a major impediment to addressing health disparities in research [11,14,15]. In the situation of unavailable individual-level SES measures, census- (or area-) level SES measures have been used as a proxy measure for individual SES measures. However, as such measures have, in and of themselves, influenced health or CVD outcomes independent of individual SES (e.g., neighborhood influence) and often result in misclassification of one’s SES [16,17,18], utilizing them as a mere proxy measure for individual-level SES may not be suitable.

Alternatively, we recently developed and validated a novel measure for individual SES based on housing features termed *HOU*sing-based *SES* measures index (*i.e.*, *HOUSES*) [19]. HOUSES is a composite index that is derived from individual housing features combined with neighborhood socioeconomic characteristics ascertained by using property address information to enumerated real property data that is available from local government assessors’ offices. HOUSES index has shown an association with various health outcomes in both children and adult [19,20,21,22].

HOUSES has not been applied to adult health outcomes such as CVD outcomes. Therefore, to assess the utility of the HOUSES index in CVD research addressing health disparities, we conducted a population-based cohort study utilizing a prospective MI cohort. Our study aims were to determine whether HOUSES can predict the risk of all-cause mortality following MI and, if associated, to identify factors that account for the differential post-MI mortality among individuals with different HOUSES (*i.e*., the pathway model) [23]. As a comparison, we assessed the relationship between educational levels as a reference SES measure and post-MI mortality.

## 2. Materials and Methods

This study was approved by the Institutional Review Boards of both Mayo Clinic and Olmsted Medical Center.

### 2.1. Study Population and Setting

Olmsted County, Minnesota, is an excellent setting to conduct population-based epidemiologic studies such as this one because medical care is primarily self-contained within the community. When patients register with any health care providers in the community at first visit (e.g., as a newborn), they are asked whether they authorize using their medical records for research. If one grants the authorization (95%) for using medical record for research, each patient is assigned a unique identifier under the auspices of the Rochester Epidemiology Project (REP) [24], which has been continuously funded and maintained since 1960. All clinical diagnoses are electronically indexed, and information from every episode of care is contained within detailed patient-based medical records; essentially all medical care settings and providers are linked. This unique longitudinal population-based resource has been the source of over 2000 publications on the epidemiology of disease [25]. Population characteristics of Olmsted County residents are similar to those of non-Hispanic white [26]. Essentially all medical care and providers are linked under the auspices of the Rochester Epidemiology Project [25,26,27].

### 2.2. Study Design

It was designed as a population-based, retrospective cohort study. We compared mortality after MI among study subjects with different SES as measured by HOUSES and educational levels as continuous and categorical (quartiles) variables. Mortality after MI was analyzed using time-to-event methods that incorporate the entire duration of follow-up for each patient. We identified factors (risk factors for MI, clinical features of MI, and therapies for MI) that account for the association between HOUSES and differential post-MI mortality, using multivariable and bivariate modeling.

### 2.3. Study Subjects

We utilized a population-based prospective cohort residing in Olmsted County, Minnesota, that had been enrolled to assess the effect of the new definition of MI on case ascertainment, conducted from 1 November 2002 to 31 May 2006. Details of the study subjects and ascertainment procedures of MI cases have been previously described [28,29]. Briefly, all Olmsted County residents who presented to an Olmsted County facility with clinical symptoms for MI and a cardiac troponin T level over 0.03 ng/mL were prospectively identified and enrolled within 12 h of the blood draw through the electronic files of the Department of Laboratory Medicine. Standardized criteria for MI were as follows: (1) chest pain; (2) electrocardiographic data using Minnesota coding; and (3) cardiac enzyme levels (cutoff value of cardiac troponin T used at Mayo Clinic; ≥0.03 ng/mL). The previous study enrolled 718 eligible Olmsted County residents. Of these 718 subjects, 696 subjects provided general research authorization for using medical records for research at the time of the present study.

### 2.4. Socioeconomic Indicators and HOUSES Index

Self-reported individual-level education status (*i.e.*, years of school completed) was collected by a demographic questionnaire. Educational years were categorized into four groups: less than 12 years, 12 years, 13–15 years, and 16 years or longer.

HOUSES is a composite index derived from housing features of real property data and address information in medical records at the time of MI event. Development and initial testing of the index were completed in both Olmsted County, Minnesota, and Jackson County, Missouri. Results from that study have been reported in previous publications [19,20,21,22]. Briefly, in formulating HOUSES, subjects’ addresses at index date of MI were geocoded. Geocoding allows for users to match study subject address to geographic reference data and real property data. Once completed, data was then spatially joined to parcels to obtain the parcel identification number or PIN. The PIN was used to join with Olmsted County’s Assessor’s real property. We applied principal component factor analysis on the basis of real property data features of housing and neighborhood SES items. Factor analysis results were pared down to four real property feature variables, including market housing value, square footage of housing unit, number of bedrooms, and number of bathrooms (neighborhood SES measures were not included in the parameters for the HOUSES index, as they were a different construct from those individual-level housing variables). We then formulated a standardized HOUSES index score by transforming the four variables to z-scores (*i.e.*, standardized index allowing comparisons across different study settings) and summing the z-scores to the HOUSES index. The higher the HOUSES z-score, the higher the SES.

### 2.5. Other Variables

The original study collected pertinent data such as cardiovascular risk factors (e.g., obesity measured by body mass index (BMI, kg/m^2^), hypertension, dyslipidemia, diabetes, smoking status, and a history of MI), MI characteristics (Killip class, ST elevation, anterior MI, and ejection fraction), comorbidity, and medications. These variables were collected from medical records and lab data obtained during the index hospitalization for MI. Smoking status was categorized as current and non-current smoking. Comorbidity was assessed by the Charlson index and analyzed categorically. Revascularization procedures included percutaneous coronary intervention and coronary artery bypass graft surgery during the index hospitalization. Ejection fraction was analyzed using three indicator variables for ≥50%, 35%–49%, and <35%.

### 2.6. All-Cause Mortality after MI

This present study utilized all-cause mortality of the original study and the details have been reported previously [28]. Briefly, ascertainment of deaths was performed by utilizing the auspices of the REP. All death certificates for Olmsted County residents are obtained every year from the county office and the Mayo Clinic registration office monitors the notice of death in the local newspapers to update the record. Finally, electronic files of death certificates are obtained from the State of Minnesota Department of Vital and Health Statistics [25]. All-cause mortality after MI was analyzed using time-to-event methods that incorporate the entire duration of follow-up for each patient.

### 2.7. Statistical Analysis

Continuous variables were summarized with means, standard deviations, medians, and ranges; categorical variables were summarized with frequency counts and percentages. HOUSES was categorized based on quartiles using the 637 patients with non-missing data as follows: (1) less than −2.0228; (2) greater than or equal to −2.0228 but less than 0.2766; (3) greater than or equal to 0.2766 but less than 1.7829; and (4) greater than or equal to 1.7829. We also analyzed HOUSES in continuous variable (z-score). Comparisons of baseline characteristics between patients with and without a HOUSES index available were evaluated using Wilcoxon rank sum, chi-square, and Fisher exact tests. Associations of baseline characteristics with quartiles of the HOUSES index and educational level group were evaluated using Spearman rank sum correlation coefficients and Cochran-Armitage trend tests. Correlations between continuous variables were evaluated using Spearman rank sum correlation coefficients. Overall survival at one year and two years after MI among subjects with different SES was estimated using the Kaplan-Meier method. We assessed whether or not the correlation between HOUSES index and post-MI mortality conforms to linearity by examining both martingale residuals from a null Cox proportional hazards regression model and observed values as well as fitting data to a smoothing spline regression [30]. Associations of SES with time to death were evaluated using Cox proportional hazards regression models and summarized with hazard ratios and 95% confidence intervals (CIs). After we assessed the association between SES and post-MI mortality, we performed multivariable Cox proportional hazards regression models to identify variables that account for the association between SES and post-MI mortality by adjusting for covariates of interest. Statistical analyses were performed using the SAS software package (SAS Institute, Cary, NC, USA). All tests were two-sided and *p*-values < 0.05 were considered statistically significant.

## 3. Results

### 3.1. Subject Characteristics

Among the 696 eligible subjects with MI, 637 (92%) were successfully geocoded to address and real property data. Comparisons of baseline characteristics, according to education levels and the HOUSES index, are summarized in Table 1. Of these subjects, 608 (95%) were white and 264 (41%) female; the mean age was 68 ± 15 years. The mean HOUSES index for these patients was 0.012 (SD 3.140; median 0.277; range −6.188 to 14.758). We were unable to geocode 59 subjects due to the following reasons: nursing home or assisted living facility (*n* = 33), P.O. box/rural route (*n* = 11), public housing (*n* = 2), and non-Olmsted County address (*n* = 13). There were no significant differences in education level, hypertension, diabetes, hyperlipidemia, BMI, aspirin, PTCA, and CABG between subjects included and excluded from the study because of unavailable HOUSES index. However, there were differences in age and the proportions of females, current smokers, and statin therapy. Educational levels were available for 659 (95%) subjects. The mean educational level was 13 years (SD 3; median 12). The Spearman’s correlation coefficient for the correlations between HOUSES and education level was 0.22 (*p* < 0.001).

### 3.2. The Association between HOUSES Index and Education Level with Mortality Post-MI

**Figure 1:**
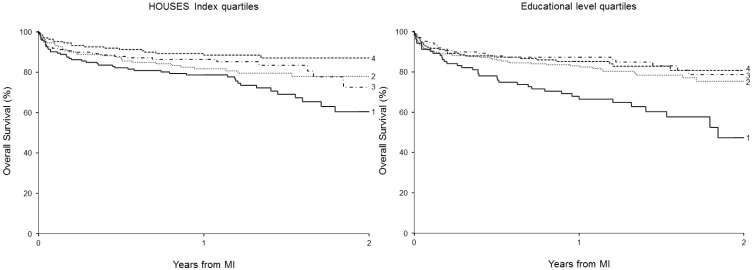
Estimated overall two-year survival rates after MI by Kaplan-Meier curves according to HOUSES index quartiles and education categories (the higher the SES categories, the higher the SES).

At last follow-up, 125 patients of the 637 patients with an available HOUSES index had died at a mean of 0.5 years following MI (median 73 days; range 0 days to 2.8 years). The estimated overall survival rate (95% CI; number of subjects at risk) following MI was 84% (81–87; 372) at one year and 75% (70–80; 56) at two years. The Kaplan-Meier curves for post-MI survival among patients with different SES measures are depicted in Figure 1.

HOUSES quartiles: 1 (lowest SES) and 4 (highest SES); educational level quartiles: 1 (lowest level of education) and 4 (highest level of education).

**Table 1:** Baseline characteristics according to education level and HOUSES index quartiles (the higher the SES categories, the higher the SES).

SES Group	Individual Education Level					HOUSES Index				
	1 (Lowest) (*n* = 100)	2 (*n* = 234)	3 (*n* = 158)	4 (Highest) (*n* = 167)	*p*	1 (Lowest) (*n* = 159)	2 (*n* = 159)	3 (*n* = 159)	4 (Highest) (*n* = 160)	*p*
Median	8	12	14	16		−4.3213	−0.7935	0.9718	3.3681	
Demographics, *n* (%)										
Age (years)	78 ± 13	68 ± 14	66 ± 15	66 ± 15	<0.001	71 ± 16	70 ± 14	66 ± 14	63 ± 14	<0.001
Female	51 (51)	114 (49)	72 (46)	55 (33)	0.001	90 (57)	65 (41)	52 (33)	57 (36)	<0.001
Caucasians	92 (92)	226 (97)	154 (97)	161 (96)	0.145	146 (92)	156 (98)	152 (96)	154 (96)	0.146
Risk factors, *n* (%)										
Prior MI	8 (8)	13 (6)	9 (6)	3 (2)	0.028	17 (11)	6 (4)	4 (3)	5 (3)	0.002
Hypertension	79 (79)	168 (72)	119 (75)	108 (65)	0.028	121 (76)	117 (74)	107 (67)	105 (66)	0.019
Diabetes	36 (36)	58 (25)	42 (27)	24 (14)	<0.001	46 (29)	38 (24)	37 (23)	33 (21)	0.092
Hyperlipidemia	56 (56)	147 (63)	105 (66)	93 (56)	0.783	97 (61)	92 (58)	100 (63)	99 (62)	0.659
Current smoker	15 (15)	63 (27)	32 (20)	15 (9)	0.008	39 (25)	30 (19)	36 (23)	24 (15)	0.081
BMI	27.9 ± 6.1	28.7 ± 6.4	28.7 ± 6.4	28.6 ± 5.7	0.379	29.0 ± 6.1	29.0 ± 6.5	28.1 ± 6.2	29.3 ± 6.2	0.684
MI characteristics and comorbidity, *n* (%)										
Killip class (*n* = 639)										
>1	40 (42)	53 (23)	33 (21)	39 (24)	0.011	54 (35)	52 (33)	30 (19)	27 (18)	<0.001
Anterior MI	48 (48)	94 (40)	52 (33)	66 (40)	0.147	67 (42)	72 (45)	61 (38)	49 (31)	0.016
ST elevation	17 (17)	44 (19)	29 (18)	42 (25)	0.097	32 (20)	29 (18)	36 (23)	34 (21)	0.587
Ejection fraction					0.070					0.015
>50	45 (57)	135 (70)	86 (68)	100 (72)		75 (60)	94 (69)	86 (67)	100 (76)	
35–49	20 (26)	40 (21)	29 (23)	27 (20)		33 (27)	33 (24)	24 (19)	23 (18)	
<35	13 (17)	17 (9)	11 (9)	11 (8)		16 (13)	10 (7)	18 (14)	8 (6)	
Comorbidity index					<0.001					<0.001
0	13 (13)	68 (29)	46 (29)	79 (47)		31 (20)	51 (32)	61 (38)	68 (43)	
1–2	37 (37)	70 (30)	69 (44)	45 (27)		57 (36)	56 (35)	51 (32)	44 (28)	
>3	50 (50)	96 (41)	43 (27)	43 (26)		71 (45)	52 (33)	47 (30)	48 (30)	
Treatment										
PTCA	37 (37)	109 (47)	79 (50)	86 (52)	0.027	67 (42)	71 (45)	86 (54)	82 (51)	0.038
CABG	8 (8)	16 (7)	16 (10)	10 (6)	0.803	3 (2)	18 (11)	13 (8)	15 (9)	0.041
Statins	62 (62)	161 (69)	111 (70)	116 (69)	0.277	102 (64)	110 (69)	122 (77)	111 (69)	0.154
β-blockers	87 (87)	208 (89)	150 (95)	151 (90)	0.176	145 (91)	147 (92)	140 (88)	143 (89)	0.348
Aspirin	90 (90)	216 (92)	149 (94)	155 (93)	0.373	149 (94)	147 (92)	148 (93)	149 (93)	0.900

Notes: Age = mean ± standard deviation; BMI = body mass index (denoted kg/m^2^ and mean ± standard deviation); MI = myocardial infarction; ST = ST segment of electrocardiogram; PTCA = percutaneous transluminal coronary angioplasty; CABG = coronary artery bypass graft surgery.

Overall, estimated post-MI survival rates at 1 and 2 years were positively correlated with HOUSES index quartile (Table 2). Similarly, survival post-MI was positively correlated with educational levels. Based on martingale residuals from a null Cox proportional hazards regression model, the correlation between HOUSES and post-MI mortality was approximately linear (data not shown). The trends analysis based on a univariate smoothing spline regression showed non-linearity; term was not statistically significant (*p* = 0.053). The results were consistent after controlling for variables included in Models 2 (*p* = 0.86) and 3 (*p* = 0.71).

**Table 2:** Survival rates at year 1 and 2 after MI according to HOUSES index and educational levels.

HOUSES	1 Year		2 Year		Education	1 Year		2 Year	
	Survival Rate (%)	95% CI	Survival Rate (%)	95% CI		Survival Rate (%)	95% CI	Survival Rate (%)	95% CI
4 (highest SES)	89	84–94	87	81–93	4 (highest SES)	85	80–91	81	74–88
3	86	81–92	72	60–87	3	87	82–93	79	70–88
2	82	76–88	78	71–85	2	83	78–88	75	68–83
1 (lowest SES)	78	72–85	60	50–72	1 (lowest SES)	68	59–78	47	33–67

Note: HOUSES index was categorized into quartiles and educational levels were categorized into four groups.

Before adjustment for covariates (Table 3: Model 1), the HOUSES index was inversely associated with mortality post-MI in a dose-response manner (*p*-value for trend <0.001). However, after adjustment for all pertinent variables (*i.e.*, Table 3: Model 3), the association was no longer significant (*p*-value for trend: 0.24). The analysis results based on HOUSES index in z-score were virtually consistent with those based on categorical variable of HOUSES: HR for HOUSES index in Model 1 was 0.91 (95%CI: 0.86–0.97, *p* = 0.0015) per an increment of one unit of HOUSES, further indicating a linear relationship between HOUSES and post-MI mortality; HR in Model 2 was 0.94 (95%CI: 0.87–1.00), *p* = 0.075; HR in Model 3 was 0.97 (95%CI: 0.91–1.03), *p* = 0.34. When we included educational levels in Model 3, the results virtually remained unchanged (data not shown).

Similarly, before adjustment for covariates (Table 3: Model 1), education level was significantly associated with mortality post-MI (*p*-value for trend < 0.001). After full adjustment (Table 3: Model 3), it was no longer significant (*p*-value for trend: 0.84).

### 3.3. Identification of Factors that Account for the Association between SES Measures and Post-MI Mortality

Given the univariate analysis results on the significant association between SES measures and post-MI mortality and no significant association after full adjustment in multivariable models, we performed multivariable Cox proportional regression models to identify individual variables that accounted for the association between SES measures and post-MI mortality. We adjusted the main association between SES measures and risk of post-MI mortality for each individual variable listed in Table 1.

**Table 3:** Multivariable cox proportional hazards models for the associations of HOUSES and educational level with two-year post-MI mortality.

Regression Models	HOUSES Index (Quartiles)					Education Level (4 Categories)				
	(Hazard Ratio, 95%CI, *p*-Value) ^b^					(Hazard Ratio, 95%CI, *p*-Value) ^b^				
	4 (ref.) (highest SES)	3	2	1 (lowest SES)	*p* ^a^	4 (ref) (highest SES)	3	2	1 (lowest SES)	*P* ^a^
Model 1 (unadjusted model)	1	1.52	1.76	2.47	<0.001	1	1.01	1.27	2.53	<0.001
		(0.85–2.73)	(1.01–3.06)	(1.46–4.19)			(0.59–1.72)	(0.79–2.03)	(1.55–4.14)	
Model 2	1	1.37	1.27	1.86	0.036	1	0.95	1.16	1.84	0.015
		(0.75–2.52)	(0.71–2.26)	(1.07–3.24)			(0.55–1.67)	(0.70–1.90)	(1.11–3.05)	
Model 3 (full model)	1	1.29	1.19	1.45	0.24	1	0.82	0.83	0.93	0.84
		(0.68–2.43)	(0.65–2.16)	(0.82–2.58)			(0.46–1.44)	(0.51–1.37)	(0.55–1.57)	

Notes: **^a^**: *p* value is statistical significance for overall test (trend) for the association between socioeconomic status and MI mortality using Cochran-Armitage trend tests; **^b^**: Hazard ratio per an increment of strata of SES group; Model 1: univariate model only including SES variable as a predictor variable; Model 2: adjusted for all variables listed in Table 1 including sex, hypertension, hyperlipidemia, smoking, BMI, Killip class, STEMI, Anterior MI and ejection fraction, PTCA, CABG, statin, beta blocker and aspirin; Model 3: adjusted for age and Charlson Comorbidity Index in addition to all variables included in Model 2.

We found that age and comorbid conditions primarily accounted for the association of SES measures with post-MI mortality, but other individual factors including MI therapy did not account for the association, as shown in Table 4. The reported parameter estimates (hazard ratios and the corresponding 95%CIs) for SES measures (HOUSES and educational levels) in Table 4 were calculated after controlling for each variable individually in a separate Cox model. For example, controlling for therapy with β-blockers compared to the highest SES (fourth HOUSES stratum), the third (HR: 1.52, 95%CI: 0.85–2.72), second (HR: 1.80, 95%CI: 1.04–3.13), and first HOUSES (lowest SES) strata (HR: 2.53, 95%CI: 1.49–4.29) had significantly increased risks of post-MI mortality (*p*-value for trend <0.001).

To confirm this finding, we performed separate multivariable regression analysis, which included all variables listed in Table 1 *except age and Charlson Comorbidity Index.* As the results are summarized in Table 3, HOUSES was independently associated with risk of post-MI mortality; compared to the highest SES (fourth HOUSES stratum), the third (HR: 1.37, 95%CI: 0.75–2.52), second (HR: 1.27, 95%CI: 0.71–2.26), and first HOUSES (lowest SES) strata (HR: 1.86, 95%CI: 1.07–3.24) had significantly increased risks of post-MI mortality (*p*-value for trend = 0.036). This was true for educational level; compared to the highest SES (fourth educational group), the third (HR: 0.95, 95%CI: 0.55–0.67), second (HR: 1.16, 95%CI: 0.70–1.90), and first (lowest SES) educational groups (HR: 1.84; 95%CI: 1.11–3.05) had increased risks of post-MI mortality (*p*-value for trend = 0.015).

## 4. Discussion

Our study results showed existence of differential mortality rates among people with different SES measured by HOUSES in Olmsted County, Minnesota, a non-inner city setting. However, HOUSES and educational levels were not independently associated with post-MI mortality, controlling for all potential factors associated with post-MI mortality. The differential mortality rates by HOUSES and educational levels were primarily accounted for by age and comorbid conditions.

In this study, we were able to geocode 92% of subjects’ address and real property data at index date to formulate the HOUSES index. Therefore, HOUSES might be able to overcome the paucity of conventional SES measures in commonly used datasets, such as administrative datasets, derived from medical records because unavailability of SES measures in commonly used datasets has been an important impediment to health disparities research [17,31]. As administrative datasets are being increasingly utilized for health service research, this could be a unique advantage of HOUSES. Importantly, HOUSES showed a significant association with post-MI mortality in a dose-response manner shown in univariate analysis of Table 3.

This relationship was supported by the similar association of educational levels with post-MI mortality in our study. Given the results, based on HOUSES index in an ordinal variable, we performed the trends analysis to determine whether the relationship between HOUSES and post-MI mortality conforms to linearity, which showed the correlation was approximately linear.

**Table 4:** Factors that accounted for the association of HOUSES and education levels with two-year post-MI mortality based on multivariable Cox models, which included corresponding SES measures and each variable listed in the Table 1. The reported parameter estimates (hazard ratios and the corresponding 95% CIs) for SES measures (HOUSES and educational levels) in the table were calculated after controlling for each variable individually in a separate Cox model.

Unadjusted HRs and 95%CI	HOUSES Index (Quartiles)					Education Level (Quartiles)				
	4 (ref)	3	2	1	*p* ^a^	4 (ref)	3	2	1	*p* ^a^
	1	1.52	1.76	2.47	<0.001	1	1.01	1.27	2.53	<0.001
		(0.85, 2.73)	(1.01, 3.06)	(1.46, 4.19)			(0.59, 1.72)	(0.79, 2.03)	(1.55, 4.14)	
**Adjusted HRs for HOUSES Index and Educational Level Controlled for each Variable Listed Below**										
**Model adjusted for:**										
Age	1	1.34	1.25	1.59	0.116	1	0.99	1.19	1.48	0.089
		(0.75, 2.40)	(0.72, 2.19)	(0.93, 2.72)			(0.58, 1.69)	(0.75, 1.91)	(0.90, 2.45)	
Comorbidity ^b^	1	1.46	1.63	1.74	0.028	1	0.75	0.83	1.33	0.186
		(0.82, 2.63)	(0.93, 2.83)	(1.03, 2.94)			(0.44, 1.29)	(0.51, 1.33)	(0.81, 2.18)	
Female	1	1.52	1.73	2.31	0.002	1	0.96	1.20	2.39	<0.001
		(0.85, 2.73)	(1.00, 3.01)	(1.35, 3.94)			(0.56, 1.64)	(0.75, 1.92)	(1.46, 3.92)	
White race	1	1.52	1.73	2.52	<0.001	1	0.99	1.27	2.58	<0.001
		(0.85, 2.73)	(0.99, 3.00)	(1.49, 4.26)			(0.58, 1.70)	(0.79, 2.02)	(1.58, 4.21)	
Hypertension	1	1.50	1.68	2.27	0.002	1	0.94	1.21	2.34	<0.001
		(0.84, 2.69)	(0.97, 2.92)	(1.34, 3.86)			(0.55, 1.62)	(0.75, 1.93)	(1.43, 3.84)	
Diabetes	1	1.50	1.75	2.35	0.001	1	0.94	1.20	2.27	0.001
		(0.84, 2.70)	(1.01, 3.04)	(1.39, 3.99)			(0.55, 1.61)	(0.75, 1.92)	(1.37, 3.75)	
Hyperlipidemia	1	1.57	1.76	2.55	<0.001	1	1.07	1.34	2.54	<0.001
		(0.88, 2.82)	(1.01, 3.05)	(1.51, 4.33)			(0.62, 1.83)	(0.84, 2.15)	(1.56, 4.15)	
Current smoker	1	1.55	1.76	2.56	<0.001	1	1.06	1.38	2.59	<0.001
		(0.87, 2.78)	(1.01, 3.06)	(1.51, 4.34)			(0.62, 1.82)	(0.86, 2.22)	(1.58, 4.23)	
BMI	1	1.36	1.61	2.38	<0.001	1	1.03	1.32	2.44	<0.001
		(0.76, 2.43)	(0.93, 2.81)	(1.40, 4.03)			(0.60, 1.76)	(0.82, 2.10)	(1.49, 3.98)	
Killip class > 1	1	1.37	1.41	2.14	0.005	1	0.96	1.19	2.23	0.003
		(0.76, 2.48)	(0.80, 2.49)	(1.26, 3.66)			(0.56, 1.66)	(0.74, 1.91)	(1.35, 3.69)	
Anterior MI	1	1.49	1.69	2.40	<0.001	1	1.02	1.25	2.44	<0.001
		(0.83, 2.67)	(0.97, 2.95)	(1.42, 4.08)			(0.59, 1.75)	(0.78, 2.00)	(1.49, 3.99)	
Ejection fraction	1	1.42	1.71	2.21	0.001	1	1.02	1.28	2.41	<0.001
		(0.79, 2.54)	(0.98, 2.97)	(1.36, 3.93)			(0.60, 1.75)	(0.80, 2.05)	(1.48, 3.95)	
Aspirin	1	1.50	1.75	2.46	<0.001	1	1.02	1.24	2.38	<0.001
		(0.84, 2.69)	(1.01, 3.04)	(1.45, 4.16)			(0.59, 1.74)	(0.78, 1.99)	(1.46, 3.89)	
β-blockers	1	1.52	1.80	2.53	<0.001	1	1.03	1.27	2.49	<0.001
		(0.85, 2.72)	(1.04, 3.13)	(1.49, 4.29)			(0.60, 1.76)	(0.79, 2.03)	(1.52, 4.07)	
Statins	1	1.68	1.78	2.39	0.001	1	1.01	1.29	2.33	<0.001
		(0.94, 3.02)	(1.02, 3.09)	(1.41, 4.05)			(0.59, 1.73)	(0.81, 2.07)	(1.42, 3.81)	
PTCA	1	1.62	1.66	2.25	0.003	1	0.94	1.17	2.04	0.004
		(0.90, 2.90)	(0.95, 2.88)	(1.33, 3.81)			(0.55, 1.61)	(0.73, 1.88)	(1.25, 3.35)	
CABG	1	1.51	1.81	2.35	0.001	1	1.04	1.28	2.62	<0.001
		(0.84, 2.72)	(1.04, 3.14)	(1.39, 3.98)			(0.60, 1.78)	(0.80, 2.04)	(1.60, 4.29)	

Notes: ^**a**^: *p* value is statistical significance for overall test (trend) for the association between socioeconomic status and MI mortality; **^b^**: Comorbidity index means Modified Charlson comorbidity index; BMI = body mass index (denoted kg/m^2^ and mean ± standard deviation) MI = myocardial infarction; ST = ST segment of electrocardiogram; PTCA = percutaneous transluminal coronary angioplasty; CABG = coronary artery bypass graft surgery.

In a recent prospective cohort study, which determined the relationships between individual socioeconomic status (e.g., income and education) and mortality rates after MI, patients with higher income showed a lower mortality rate than those with a lower income (unadjusted HR, 0.45; 95% CI, 0.35–0.57, *p* < 0.001) [15]. In addition, Mehta *et al.*, reported in a study that evaluated 11,326 patients with ST elevation MI that one-year mortality was inversely related to individual educational level, and years of education remained independently related to mortality following MI between day 8 and 1 year (HR per year of increase in education; 0.96; 95% CI: 0.94–0.98, *p* < 0.001) [11].

Despite the significant association of HOUSES with post-MI mortality, there was not a strong correlation between HOUSES and educational levels. This finding might not necessarily be unexpected given the reported modest correlation between education and income (*r* = 0.33) [17]. These findings suggest that HOUSES has little redundancy in measuring one’s SES by other SES measures and may provide supplementary information in addition to other SES measures. In support of this, Smith [32,33] and others showed that household asset-based SES measures, especially housing features (e.g., home ownership) were associated with various health outcomes [34,35,36,37,38,39]. Along these lines, it would be worth examining the role of neighborhood SES in post-MI mortality using a multi-level analysis in the future. At any rate, since HOUSES has not been applied to CVD research and MI affects a significant proportion of Americans, HOUSES could help researchers, clinicians, and policymakers address health disparities in CVD through enhancing health disparities research.

Although the association between HOUSES index and educational level with post-MI mortality was significant in unadjusted analysis, after full adjustment of all pertinent variables, the HOUSES index and education level did not independently predict post-MI mortality. The univariate analysis results (the association between SES measures and post-MI mortality) and these multivariable analysis findings prompt us to identify which factors potentially account for the association between SES measures and post-MI mortality (the pathway model) [23]. We found that none of the traditional risk factors for MI including post-MI therapies, clinical features of MI, and risk factors for MI accounted for the association of HOUSES and educational levels with post-MI mortality, but age and comorbid conditions as shown in Table 4 did. Our multivariable regression analysis confirmed these findings (Table 3: Model 2): both HOUSES and educational levels predicted post-MI mortality independent of all traditional risk factors for MI except age and Charlson Comorbidity Index. At the same time, based on the full model (Model 3), both HOUSES and educational levels lacked the independent effect on post-MI mortality. Because of a potential concern about over-adjustment for comorbidity, which can be in a causal pathway between SES and mortality, we re-ran the analysis without comorbidity, but the results did not change significantly (data not shown). While the effect of HOUSES on post-MI mortality was only attenuated, that of educational levels on post-MI mortality was changed in Model 3 (*i.e.*, the direction of the association).

A recent prospective study showed that adjustment of age and cardiovascular risk factors significantly attenuated the impact of income on post-MI mortality (from HR of 0.45 to 0.77) [15], but we were unable to find similar study results in the literature to compare with ours. These results potentially suggest that to reduce the gap in differential post-MI mortality rates among people with different SES, efforts should include prevention and management of non-cardiac comorbid conditions [40,41] associated with lower SES, and assessment of medical needs should take into account patients’ socioeconomic context, especially in an aging population with declining SES. Mediation analysis, as a follow-up study for our current results, needs to be considered in the future. In addressing health disparities, several conceptual models have been proposed (*i.e.*, the genetic model, the fundamental cause model, the pathway model, and the interaction model) [23]. While these conceptual models need to be investigated in understanding health disparities, our study results could support the pathway model, which emphasizes the understanding of pathways underlying health disparities [23]. A major strength of this study is a population-based study design based on prospectively identified MI cases with a longitudinal follow up. Also, our study setting is a self-contained health care environment with availability of comprehensive medical records of nearly all Olmsted County residents under the auspices of the REP. The HOUSES index has unique advantages: it is not an aggregated measure but an individual-level SES measure; it is based on objective measures with Assessor’s data and not self-reported; availability of electronic real property data allows formulation of HOUSES for a large-scale data through geocoding by using Geographic Information System program. Therefore, the data and procedures needed for formulating HOUSES are relatively simple and readily available (1) address information, which is always available in medical records (or medical record-derived administrative data); (2) the Assessor’s data, which are electronically and publicly available in most counties in the US; and (3) geocoding using GIS (matching the Assessor’s data and address), which is currently a routine procedure.

There are limitations to be considered in the interpretation of our study results. Factors related to SES data, such as stress, lifestyle, diet. *etc.*, were unavailable in our study. Our only parameter for correlation was educational-level data. Future studies may need to address these limitations and identify other SES data linkages. The sample size of this study is relatively modest, but we were able to address the study aims with the available sample size. Study subjects were predominantly Caucasian, which could limit generalizability of study findings in other settings. However, it may minimize the confounding effect of ethnicity that is often intertwined with SES. We were able to geocode 92% of all subjects, but there were no significant differences in educational level (*i.e.*, SES) between subjects included and those excluded from the study. Although there is a possibility of transfer bias (selection bias), given the fairly consistent findings between HOUSES and educational levels, we believe transfer bias is unlikely to account for our study findings entirely. Although our original analysis to formulate the HOUSES index included the tenure status (ownership status), as our community has a high ownership rate (about 75%), the tenure status did not play a major role in predicting housing-based SES status.

## 5. Conclusions

Although HOUSES is associated with post-MI mortality, the differential mortality rates by HOUSES were primarily accounted for by age and comorbid conditions. As the prerequisite for formulating HOUSES is readily available, HOUSES will be useful for epidemiological research concerning CVD outcomes among adults when conventional SES measures are unavailable in commonly used datasets. However, further studies are needed to determine generalizability of HOUSES to other study settings. Also, health disparities in post-MI mortality need to be further studied under different conceptual models for health disparities, and HOUSES may enhance this endeavor.
